# A first look at sea-lavenders genomics – can genome wide SNP information tip the scales of controversy in the *Limonium vulgare* species complex?

**DOI:** 10.1186/s12870-022-03974-2

**Published:** 2023-01-16

**Authors:** Francisco Pina-Martins, Ana D. Caperta, Sofia I. R. Conceição, Vera L. Nunes, Isabel Marques, Octávio S. Paulo

**Affiliations:** 1 cE3c - Centre for Ecology, Evolution and Environmental Changes & CHANGE - Global Change and Sustainability Institute, Departamento de Biologia Animal Faculdade de Ciências, Universidade de Lisboa, Campo Grande, 1749-016 Lisbon, Portugal; 2 LEAF—Linking Landscape, Environment, Agriculture and Food, Associated Laboratory TERRA, Instituto Superior de Agronomia (ISA), Universidade de Lisboa, Tapada da Ajuda, 1349-017 Lisbon, Portugal; 3 LASIGE Computer Science and Engineering Research Centre, Faculdade de Ciências, Universidade de Lisboa, 1749-016 Lisbon, Portugal; 4 Forest Research Centre (CEF) & Associated Laboratory TERRA, Instituto Superior de Agronomia (ISA), Universidade de Lisboa, 1349-017 Lisbon, Portugal

**Keywords:** Atlantic Europe, Genotyping by sequencing, Polyploidy, Western Mediterranean, *Limonium vulgare*, *L. maritimum*

## Abstract

**Background:**

Sea-lavenders (*Limonium* Mill., Plumbaginaceae) are a cosmopolitan group of diploid and polyploid plants often adapted to extreme saline environments, with a mostly Tethyan distribution, occurring in the Mediterranean, Irano-Turanian, Euro-Siberian and in the New World. The halophylic *Limonium vulgare* polyploid complex in particular, presents a large distribution throughout extreme salt-marsh habitats and shows little morphological but high taximetric variation, frequently blurring species delimitation. In this work we pursue three main goals: assert whether SNP data from polyploid individuals has the resolution to distinguish the seven sampled species, to better understand how genetically structured *Limonium vulgare* is, and attempt to identify specific molecular mechanisms for the differentiation between *L. maritimum* and *L. vulgare*. For this purpose, 95 individuals were genotyped using Genotyping by Sequencing (GBS), which were assembled as two independent datasets using ipyrad. All analyses performed downstream of assembly were fully automated. Phylogenetic inference, PCA, and admixture plots were used to infer answers to the study’s main goals.

**Results:**

Close to 10,000 SNPs were obtained for each dataset. Phylogenetic analyses reveal that polyploid data can be used to infer species relationships. Population structure analyses suggest a genetically structured *L. vulgare*. A set of 34 SNPs were found to be fully segregated between *L. vulgare* and *L. maritimum*, two of which are potentially linked to proteins that might be involved in the speciation process.

**Conclusion:**

Despite polyploid data analyses shortcomings, GBS generated SNPs have the resolution to discern all seven included species. *Limonium vulgare* revealed pronounced genetic structure along a geographical north-south cline. *L. maritimum* always appears as a distinct genetic entity. Segregated SNPs between *L. vulgare* and *L. maritimum* indicate salinity response and morphological trait control genes as potentially interesting to follow up for studying these species’ divergence process.

**Supplementary Information:**

The online version contains supplementary material available at 10.1186/s12870-022-03974-2.

## Background

Sea-lavenders (*Limonium* Mill., Plumbaginaceae) are cosmopolitan organisms, that survive in environments containing significant concentrations of soluble salt (halophytes) in which they grow, such as, rocky cliffs, and salt marshes that can be inundated with seawater during high tide. Many of these halophytes show a high level of endemicity since they grow in very specialized microhabitats that occupy small areas, usually exposed to intense anthropogenic disturbance [1–4].

This plant group has a mostly Tethyan distribution, occurring in the Mediterranean, Irano-Turanian and Euro-Siberian regions. The *Limonium vulgare* complex is represented by species such as *Limonium vulgare* sensu *lato* (west Atlantic, tetraploid), *Limonium maritimum* (west Atlantic, tetraploid), *Limonium humile* (west Atlantic, hexaploid [5]) or *Limonium narbonense* (west Mediterranean, tetraploid) [6, 7] but also includes New World native species, such as *Limonium carolinianum* (east Atlantic, tetraploid [8]), *Limonium californicum* (western Pacific, diploid [9]) and *Limonium brasiliense* (east Atlantic, unknown ploidy) [10]. *Limonium* from subfamily *Limonioideae (= Staticoideae* [10]) has a high enough incidence of polyploidy [11–13] and several taxonomical complex groups have been identified that include ca. 600 species [6, 7, 11, 12, 14–16]. Species from *L. vulgare* complex present diverse breeding systems such as selfing (*L. californicum*, *L. carolinianum*, *L. humile*), outcrossing (*L. brasiliense*, *L. narbonense*, *L. vulgare*) and probable apomixis (agamospermy, asexual reproduction through seeds; *L. maritimum*) [7, 9, 12, 17–19]. The complex’s nominal species, *L. vulgare*, is particularly interesting as a study subject, since due to its distribution, it is subject to a wide spectrum of environmental conditions and is sympatric to other closely related species such as *L. maritimum*, or *L. humile* [7, 12, 20, 21].

**Fig. 1:**
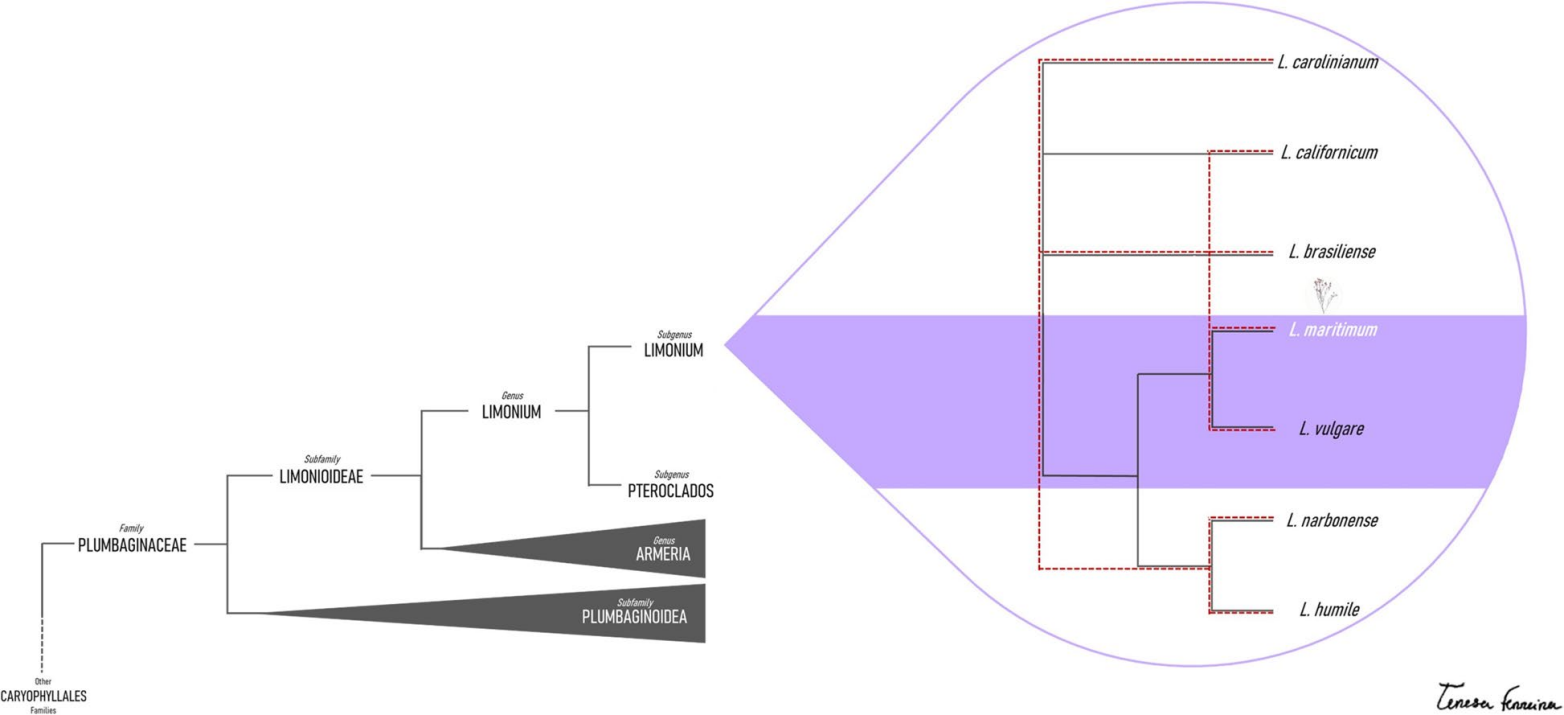
Conceptual cladogram showing phylogenetic relationships found between species of the polyploid *Limonium vulgare* complex (Plumbaginaceae) based on previous genetic studies on these species (Malekmohammadi, Akhani & Borsch, 2017; Koutroumpa et al., 2018; Róis et al., 2018). The continuous black line represents relationships found in Malekmohammadi, Akhani & Borsch, (2017) and the red dashed line represents those reported in Koutroumpa et al., (2018). Relationships between *L. vulgare* and *L. maritimum* are based in Róis et al. (2018)

Phylogenetic relationships between *Limonioideae* subfamily members are intricate and complex, but thoroughly explored using plastidial and nuclear markers in [10, 22–25]. Relationships between *L. vulgare*, *L. narbonense*, *L. humile*, *L. carolinianum, L. brasiliense* and *L. californicum* are not yet entirely clear, with different markers proposing different optimal trees, but always segregating the mentioned species [10, 22–25]. Some studies, however, are unable to show a phylogenetic differentiation between *L. maritimum* and *L. vulgare* [25]. This not only triggers the question of how well differentiated these two species are on a genomic level, but also of what could have caused this segregation. Figure 1 conceptually summarizes the current known phylogenetic relationships and uncertainties among species within the *L. vulgare* complex.

Relatively recent technological advances – “reduced representation libraries” (RRLs) such as “Genotyping by Sequencing” (GBS) [26] or “Restriction site-Associated DNA Sequencing” (RAD-Seq) [27] are able to prove solutions to both presented problems in a cost-effective way [28]. Resulting markers from these techniques – “Single Nucleotide Polymorphisms” (SNPs), can be used to both resolve difficult phylogenies [29], and provide insights regarding population genetics [30]. Use of these techniques, however, is not without its challenges, especially when applied to polyploid individuals [31, 32]. Although analyses pipelines for polyploid individuals do exist, each has limitations that prevent their generalized application [31]. Further downstream, polyploidy presents more data analyses barriers, for example regarding genotype assessment, association studies or even with F_ST_ based methods [31, 33]. Nonetheless, polyploid groups provide some of the most interesting organisms to study from an evolutionary point of view, especially regarding speciation dynamics [13, 34]. As such, this study’s first objective is to establish whether SNP data from polyploid individuals are able to differentiate the sampled species. For that, GBS was used to obtain SNPs from a set of 95 individuals of different *Limonium* species: *L. vulgare*, *L. maritimum*, *L. narbonense*, *L. humile*, *L. carolinianum, L. brasiliense* and *L. californicum*. Once this is demonstrated, three main follow-up questions ensue: a) Which phylogenetic hypothesis from Fig. 1 is supported by the GBS data, b) How genetically structured is *L. vulgare* across its distribution range, and c) Can SNPs that segregate the closely related species *L. maritimum* and *L. vulgare* be found in genes that help explain the species’ differentiation?

Answering these questions will contribute to better understanding the controversial phylogenetic relationships between the studied *Limonium* complex species, especially regarding the relationship between *L. vulgare* and *L. maritimum*, as well as providing a comprehensive overview of *L. vulgare*’s population genetic structure.

## Results

The obtained raw data FASTQ files contained ~ 408 M reads in each pair file. After demultiplexing in 96 samples (95 plant samples plus a “blank” negative control), 9,308,503 pairs were lost due to barcode mismatch and 19,372 were assigned to the “blank” barcode, which left ~ 398 M read pairs distributed among all 95 samples and used in the “species” dataset. For the “lvu_lma” dataset ~ 263 M read pairs from 65 samples were used.

Each above-mentioned set of reads was independently assembled with ipyrad, resulting in 111,855 SNPs in 16,520 contigs for the “species” dataset, and 439,831 SNPs in 142,032 contigs for the “lvu_lma” dataset.

After filtering the resulting assemblies, 10,720, and 10,485 SNPs (in the same number of contigs) represented across 86, and 57 individuals remained in the “species”, and “lvu_lma” datasets respectively.

### Phylogenetic analysis

**Fig. 2:**
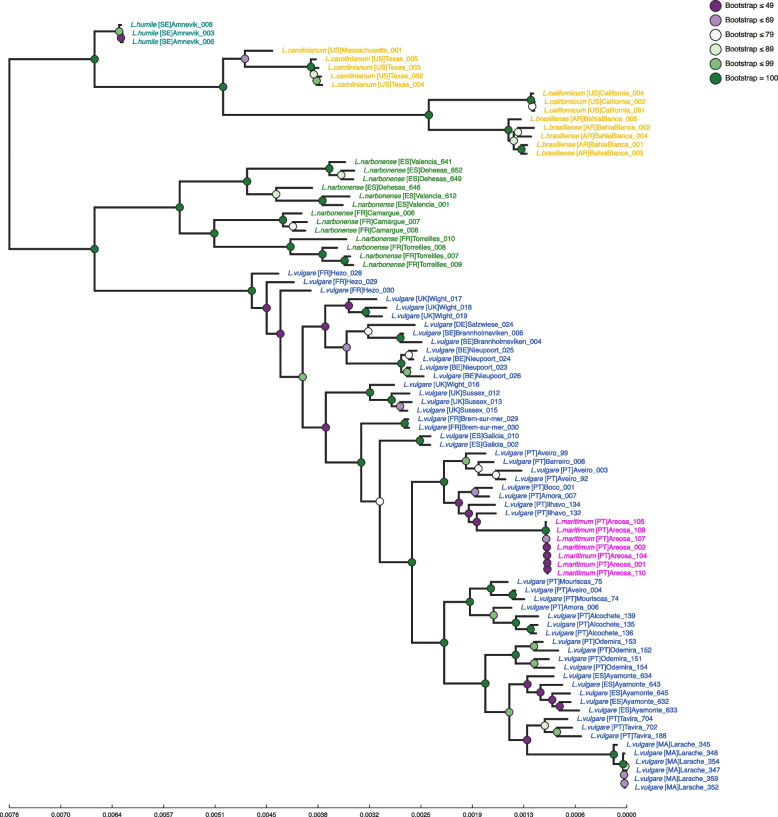
Maximum Likelihood tree of *Limonium* samples analysed for this study. New world species are represented in Yellow, *L. narbonense* in green, *L. humile* in teal, *L. maritimum* in magenta, and *L. vulgare* in blue. Circles in each node represent tree support in the form of bootstrap values, colour coded as per the top right corner information. Country of sample origin is stated as ISO 3166-1 alpha-2 codes in brackets after the species’ name

Phylogenetic analyses were performed on 86 samples comprising a 1,950,198 base-pairs alignment, with 257,083 unique patterns (number of unique columns). The resulting tree can be seen in Fig. 2. These results indicate, first and foremost, that the analysed dataset is able to distinguish all species, with each forming its own monophyletic group, except the *L. vulgare* group, which contains *L. maritimum*, which consequently makes it a paraphyletic species. Furthermore, this distinction is always supported by very high bootstrap support values (always 100).

Two major, well-supported clades can be seen in this tree. The first one comprises all *L. humile* and New World species individuals. Each of these species forms its own, well differentiated clade, with *L. humile* as a basal group to the American species, which reveal *L. carolinianum* as basal to *L. brasiliense* and *L. californicum* clades. The second clade is sub divided in two groups. One comprising *L. narbonense* samples, and another comprising *L. vulgare* and *L. maritimum* individuals. The *L. narbonense* clade has a highly supported geographic structure, clearly separating samples from France and Spain. The *L. vulgare* group is also geographically structured, with well-supported sub-clades, but relationships between them are not as strongly supported as in previously observed groups. Phylogenetic reconstruction shows a series of ladder-like clades (with varying degrees of support) comprised of individuals from *L. vulgare*’s northern range: Sweden, United Kingdom, France, Belgium, Germany, and Northern Spain (Galicia). After this group, a single clade is formed comprised of samples from the species’ southern range (and *L. maritimum*) samples.

It is worth noting that in southern samples, the *L. maritimum* clade is grouped within a monophyletic clade of *L. vulgare* samples from the same geographic region, but at considerable distance from the latter. A similar pattern can also be observed regarding *L. vulgare* samples from *Larache* (Morocco), whose clade is also at a considerable distance from its sister taxa (*Ayamonte*).

### Ordination and clustering analyses

**Fig. 3:**
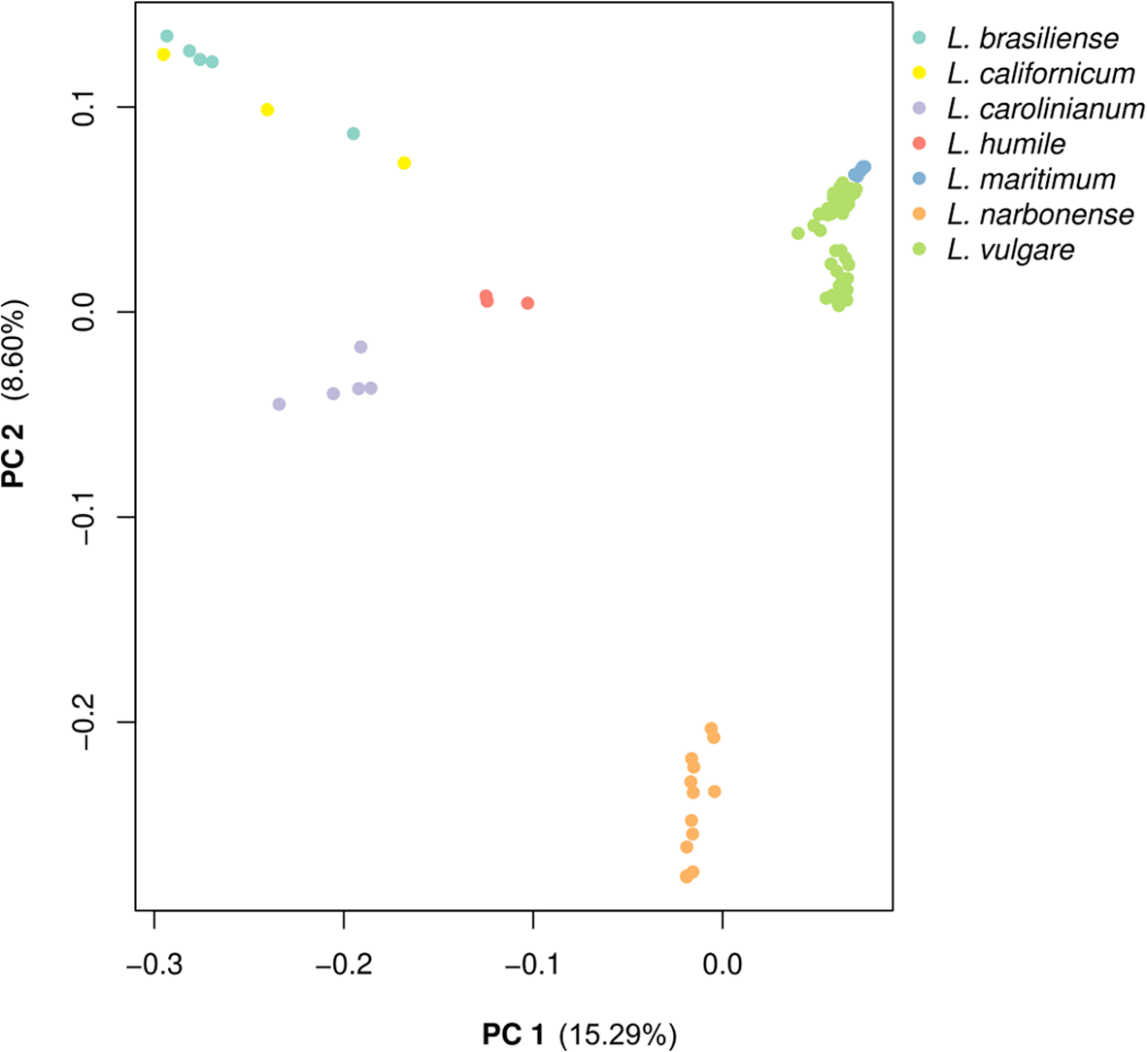
PCA of the “species” dataset. Each species is represented in a different colour. The percentage of variance explained is 15.29 and 8.60% for Principal Component 1 and 2 respectively

**Fig. 4:**
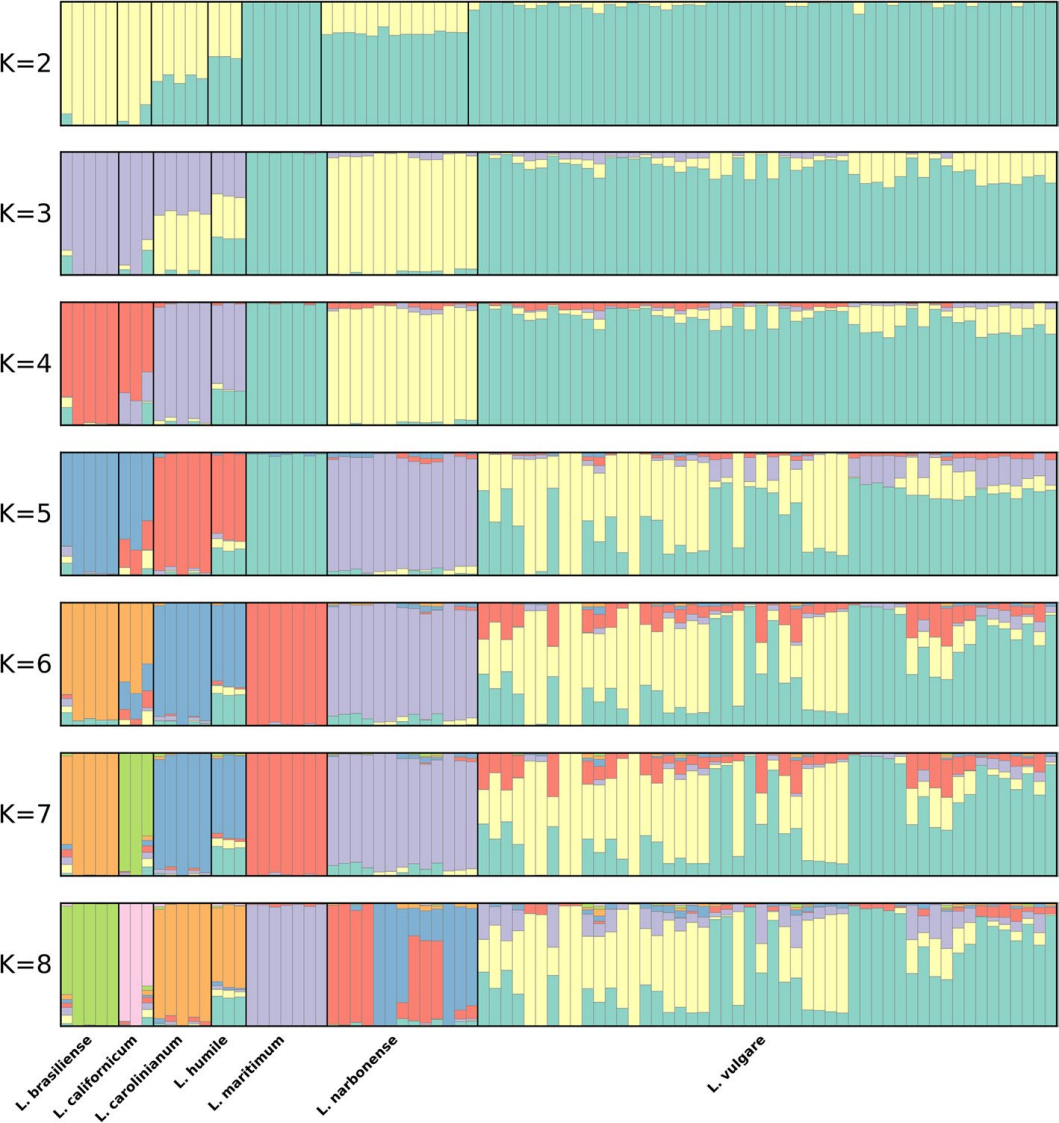
Clustering analyses results of the “species” dataset based on ALStructure for values of K between 2 and 8. Each colour represents a different cluster

PCA results of the “species” dataset (Fig. 3) reveal that 10,720 SNPs segregate each of the recognized species, similar to phylogenetic analysis results. This PCA further revealed *L. vulgare* and *L. maritimum* as the closest species, with *L. narbonense* as the closest species to the previous two. Further away from all other species it is possible to find *L brasiliense* and *L. californicum*. It is worth noting that Principal Component (PC) 1 and 2 explain a relatively high amount of variance: 15.29 and 8.60% respectively. Results of the clustering analyses performed by ALStructure can be seen in Fig. 4. The observed pattern is concordant with both PCA and phylogenetic analysis results, since each species can be identified by a different cluster (given a large enough K value). It is worth pointing out, however, that *L. humile* and *L. carolinianum* are always similarly classified, even though for higher values of K (K > 5), *L. vulgare* and *L. narbonense* are split in multiple intra-specific clusters.

**Fig. 5:**
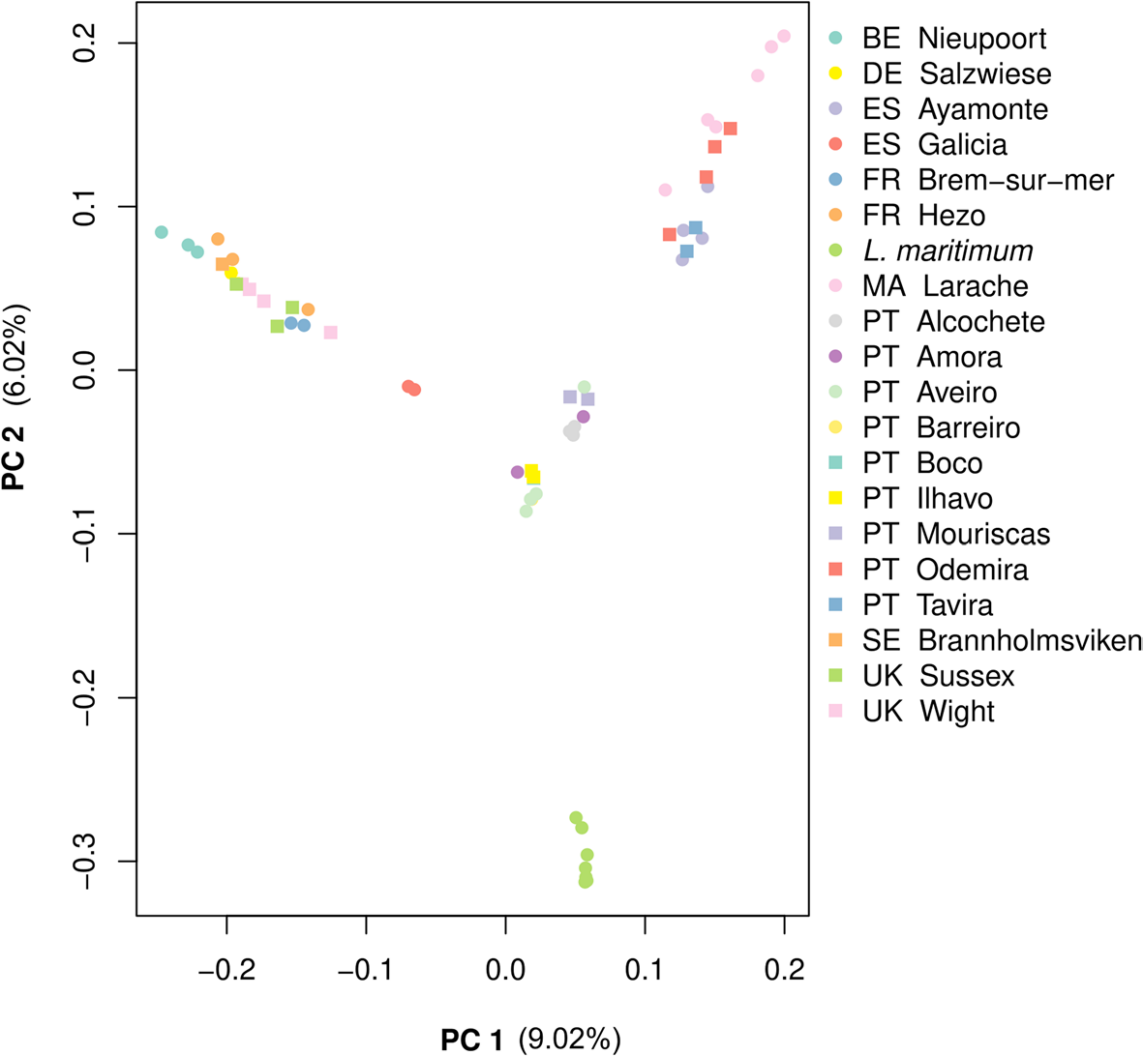
PCA of the “lvu_lma” dataset. The percentage of variance explained is 9.02 and 6.02% for Principal Component 1 and 2 respectively. The two capital letters before each location in the legend stand for the ISO 3166-1 alpha-2 codes from where *L. vulgare* samples were collected

**Fig. 6:**
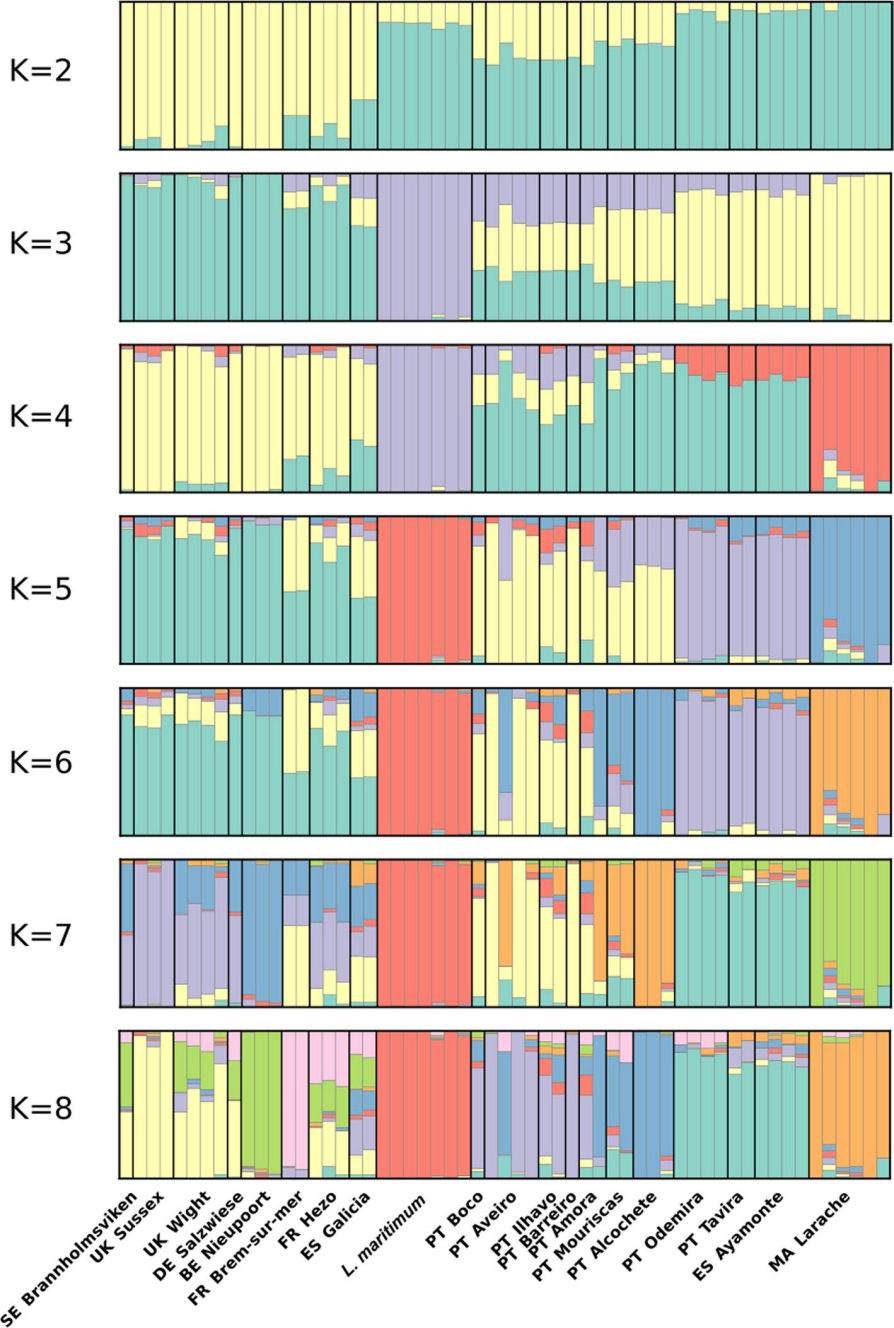
Clustering analyses results of the “lvu_lma” dataset based on ALStructure for values of K between 2 and 8. Each colour represents a different cluster. The two capital letters before each location in the legend stand for the ISO 3166-1 alpha-2 codes from where *L. vulgare* samples were collected

PCA results of the “lvu_lma” dataset (Fig. 5) reveal three groups – one with *L. maritimum* individuals, another one with southern *L. vulgare* samples, and one other with northern *L. vulgare* samples. Segregation between northern and southern individuals occurs along PC1, whereas segregation within groups occurs mostly along PC2 (9.02 and 6.02% variance explained respectively), suggesting that different groups of SNPs are responsible for the observed pattern. Within southern *L. vulgare* samples, it is further possible to identify two geographically distinct subgroups – one with samples from northern and centre Portugal, and another with southern Portugal, southern Spain and Moroccan samples. In the northern *L. vulgare* samples group, Galician samples appear relatively isolated. ALStructure results (Fig. 6) are generally compatible with PCA patterns, displaying the same clustering gradient between northern and southern *L. vulgare* individuals, and with *L. maritimum* samples isolated from *L. vulgare* in their own cluster from K ≥ 3. It is worth highlighting that the two southern samples’ clusters can be observed in both the PCA and ALStructure (for high enough values of K) plots, but the Galician samples’ isolation is not discriminated in ALStructure. Furthermore, ALStructure results reveal a cluster composed exclusively of samples from *Larache* for every K ≥ 4. This pattern is not as evident in the PCA, but is also present and well-supported in the phylogenetic tree (Fig. 2).

#### *L. vulgare* and *L. maritimum* differentiation

Thirty-four SNPs were found to be fully segregated between *L. vulgare* and *L. maritimum*. Of these, three were matched to *L. bicolor*’s transcriptome sequences (Supplementary Table 3). In total, eight sequences were matched to UniParc peptides, but two of these hits were discarded since they did not match plant peptides, resulting in six hits that are potentially worth further studying. No relevant matches were found with NCBI’s “nt” database.

Locus 984 was matched to uncharacterized proteins, but in the vicinity to reverse transcriptase proteins, which are associated with retrotransposons in plants [35].

Locus 3813 was matched to “Tetratricopeptide-like helical domain superfamily” proteins, which is a structural motif present in a wide range of proteins. In this case, it was found associated with “Pentatricopeptide repeat”, which is involved in multiple aspects of RNA metabolism [36].

Locus 4214 (GBRK01021417.1) was matched to a “TCP transcription factor family”, which is known to be involved in the evolution of key morphological traits, including plant growth and development [37], including flower development [38, 39].

Locus 12,565 was matched to an “Endonuclease/exonuclease/phosphatase superfamily” protein, which is known to be involved in intracellular signalling [40].

Locus 46,709 (GBRK01023424.1) was matched to an “MFS transporter superfamily” protein. These are known to play important roles as transport proteins, essential for the movement of a wide range of substrates across biomembranes [41], and are known to be linked to salinity tolerance in plants [42].

Locus 95,053 (GBRK01036747.1) was matched to a “Zinc finger, PHD-finger” protein. These proteins modify chromatin as well as mediate molecular interactions in gene transcription, which can work as epigenome readers that can influence gene expression [43].

## Discussion

Species of *L.* sect. *Limonium* subsect. *Genuinae* (Boissier 1848) are among the most taxonomically difficult groups in *Limonium* due to the occurrence of intermediate characters [7, 10, 21]. Previous phylogenetic works are not in accordance with this group’s relationships [10, 22–25] (Fig. 1). The present work attempts to bring new insights into the group’s relationships resorting to high throughput sequencing technology. It is worth highlighting that *L. vulgare*, *L. maritimum*, *L. narbonense* and *L. carolinianum* are tetraploid, whereas *L. californicum* is diploid and *L. humile* is hexaploid [5, 8, 20, 21]. This presented additional data analyses challenges, since most methods (both before and after SNP calling) are designed for diploid, or sometimes haploid species [44].

The used GBS dataset was “assembled” into two separate SNP datasets, each of which optimized for reaching this work’s proposed objectives. In both cases, conservative approaches to SNP filtering were used, which caused a near 10-fold decrease in number of “raw” to filtered SNPs on each dataset. Of these, the most impactful is the linkage disequilibrium mitigation strategy, which discards all but one SNP from each contig to avoid bias [45]. A close second is the decision to remove all loci with a minimum allele frequency below 2 alleles [46]. The approach to handling missing data, which is limited to a maximum of 20% per locus, a strategy devised from [47] further reduced the number of resulting SNPs. Despite these stringent filtering strategies, the number of obtained SNPs in each dataset is similar to those found in other comparable studies [48, 49]. It is worth noting, however, that using less conservative missing data thresholds did have noticeable impact. Leaving individuals with high amounts of missing data would cause them to be grouped in unexpected positions, while leaving SNPs with much missing data would substantially decrease the PCA’s amount explained variance.

### Does the GBS data from polyploid individuals have the resolution to distinguish the sampled species?

To provide an answer for the first question proposed in this work, the “species” dataset was used. All performed analyses, (Phylogenetics, PCA and clustering analyses) seem to indicate that indeed the obtained GBS data has the resolution to distinguish sampled individuals at species level. Results seem to corroborate what most of the literature already sates – *L. vulgare* and *L. maritimum* are genetically very close, *L maritimum* seems to have originated from *L. vulgare* [7, 12, 25], and *L. narbonense* is sister taxa to *L. vulgare* [10, 22–25]. On the other hand, data suggests that *L. californicum* and *L. brasiliense* are closer to each other than to *L. carolinianum*, which is compatible with results from [24], but not from [10], where this relationship is missing. This group is in turn closer to *L. humile* than to any other included species. Although this grouping is very strongly supported, it is important to highlight that a previous study [24] positioned *L. humile* closer to *L. narbonense* than to *L. carolinianum*. *Limonium humile* and *L. carolinianum* are both Atlantic species [9, 10, 17], and share traits not common to *L. narbonense* (which has a southwestern Mediterranean distribution [6, 7, 16, 50]) such as the selfing breeding system [9, 17, 21, 51]. However, *L. humile* is hexaploid, while the other two species are tetraploid, and this difference is known to bias clustering patterns [44], which might explain the observed arrangement. As such, this particular relationship should be interpreted with caution, and confirmed with more studies, specifically resorting to long read whole genome sequencing approaches.

### How genetically structured is *L. vulgare* across its distribution range?

Analyses of the “lvu_lma” dataset provide some insights regarding *L. vulgare*’s population structure. The most evident segregation in this dataset is between samples from the northern (Sweden, United Kingdom, Germany, Belgium, France and northern Spain) and southern (Portugal, southern Spain and Morocco) distribution ranges. The gradient like clustering pattern is especially evident Fig. 6’s K = 2 plot, which suggests some degree of isolation by distance. Intermediate K values reveal a fragmented southern group, with a differentiated *Larache* (Morocco) cluster and even a third cluster comprised of *Odemira*, *Tavira* and *Ayamonte* individuals (southern Iberian Peninsula). Only for very high values of K (7 and 8) can evidence of fragmentation in the northern group be found, with samples from *Nieupoort* (Belgium) and *Brem-Sur-Mer* (France) each forming independent clusters. These results suggest that *L. vulgare* is likely genetically structured throughout its distribution range. This is contrasting with morphometric [21] and taximetric [7] studies of *L. vulgare* complex, where no correlation is found between taximetry and geographic distribution.

Future studies can explore these insights to understand the reasons for this structure, using local adaptation detection and genomic offsets [52]. This will require a specifically designed sampling strategy, comprised of both more locations and more samples per location than those found in this study.

### Differentiation between *L. maritimum* and *L. vulgare*

Previous works have identified *L. maritimum* and *L. vulgare* as distinct, albeit close species [7, 25]. Both phylogenetic and population genetics results in this work seem to support this hypothesis, but do not provide context on the genetic basis for this differentiation. Fully segregated SNPs between these species, however, do provide some clues in this regard. Although only a very small percentage of the analysed SNPs were completely segregated between both species (34 of 10,485), it was possible to match some of their flanking regions to known proteins.

Only six of the 34 segregated SNPs were mapped to known protein regions. Of these, four were matched to “house-keeping” genes (Loci 984, 3813, 12,565 and 95,053), which provide no indication regarding the differentiation’s context. However, two loci (Loci 4214, and 46,709) were matched to proteins with functions which may help to pinpoint what could have caused these two species to diverge. Although locus 46,709, which can be linked to salinity response, is a very good candidate for follow-up studying in these halophyte species, as a differential tolerance to salinity could help explain the observed divergence, it is locus 4214, linked to TCP transcription factors, that provides the most important clue to explain the mentioned divergence. The current speciation hypothesis, proposed in [7] is based on the fact that *L. vulgare* presents heterostylic flowers and dimorphic populations with self-sterile pollen-stigma combinations (A or B), associated with sexual reproduction (outcrossing) [7, 17, 21, 53–55], whereas *L. maritimum* specimens show exclusively homostylic flowers with a unique self-incompatible pollen-stigma morph (B) and very low fertility [7], probably associated to apomixis (agamospermy, asexual reproduction through seeds). TCP transcription factors are involved in various flower development aspects, including shape and symmetry [38, 56]. These genes’ activities are also essential for leaf development [57] and ovule development [58]. Other key biological process is in hormone regulation of auxin homeostasis (auxin perception and response) in the gynoecium by binding the IAA3 promoter in *Arabidopsis* [59]. Further, changes in TCP expression result in male and female sterility [56]. In a recent study on floral sRNAs originated from apomictic and sexual genotypes of *Paspalum notatum* transcription factors from the TCP subfamily is affected in apomictic ovules [60].

The segregation in locus 4214 between these species, which is linked to flower shape morphology supports this hypothesis, by providing a genetic basis to the morphological observations. Although this link does not confirm causality, further studying these species’ TCP transcription factor proteins and reproductive systems, through wet-lab validation methods may yield further insights into this subject which is a strong candidate for follow-up studies.

## Conclusions

In this work, three main biological questions were addressed, which provide new insights on part of the *Limonium* complex evolutionary history, as well as intra and interspecific relationships.

Results demonstrate that genome wide SNP data such as the one used here can match those of sequence data regarding species discrimination within the complex. Although this has been widely demonstrated before [61], in this particular case, polyploidy could have biased the results, which meant that validation was in order. At population level, polyploidy also played a role, limiting the type of analyses that can be accurately performed, such as *F s*tatistics-based methods [44]. As such, alternative methods, like the model-free approach from ALStructure, were found to answer the proposed questions with a polyploid dataset.

This study is, to date, using the largest data matrix to infer phylogenetic relationships in the *L. vulgare* complex. As such, it is able to provide detailed interspecific relationships with unprecedented support levels, while also confirming *L. maritimum* as a genetically distinct entity from *L. vulgare*, making the latter a paraphyletic species.

At an intra-specific level, this study has two main highlights. *L. vulgare* is revealed to be a genetically structured species, with clear northern and southern clusters. Furthermore, samples from *Larache* (Morocco), reveal a differentiation pattern from other individuals, somewhat similar to *L. maritimum*’s divergence pattern.

Both inter and intra-specific revealed results are worth further exploring in follow-up studies with more fine-grained approaches, such as WGS, and/or with more comprehensive sampling strategies.

Regarding *L. vulgare* and *L. maritimum* divergence, this study’s findings provide novel evidence supporting the hypothesis proposed in [7] based on morphological traits. These results highlight the potential role of TCP transcription factors in these species’ divergence, and suggest a starting place to further study these two close species’ divergence.

## Methods

### Biological samples

**Fig. 7:**
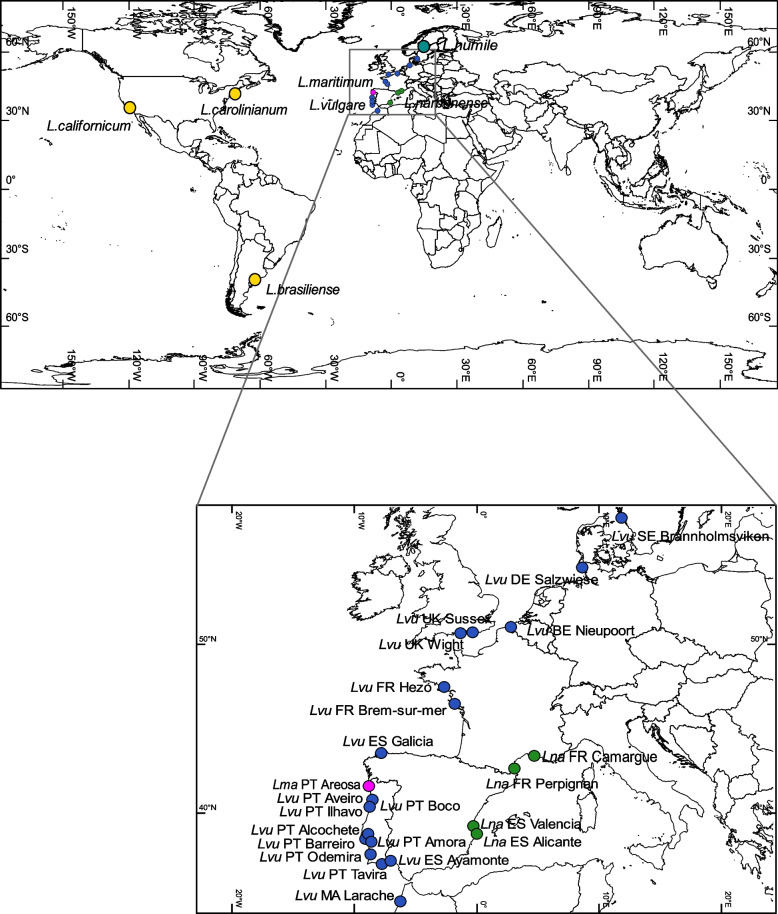
A map of the sites sampled for this work. In European samples the first part of the label represents the species: “Lvu” - *L. vulgare*; “Lma” - *L. maritimum*; and “Lna” - *L. narbonense*. The second part represents the country of origin according to ISO 3166. The third part is the name of the location where samples were collected

Leaf material was obtained from 95 individuals: 33 plants were sampled in natural populations (Supplementary Table 1), and 62 were originated from seeds planted and germinated at “Instituto Superior de Agronomia” (ISA). Of these seeds, 25 were field collected (Supplementary Table 1), and 37 were requested from seed banks (Supplementary Table 2). A total of 30 sites were sampled, as shown in Fig. 7.

Formal sample identification was undertaken by Ana D. Caperta following the treatments of [6, 7, 62–64]. Voucher specimens were deposited in the Herbarium João de Carvalho e Vasconcellos (LISI), Instituto Superior de Agronomia, Portugal (Supplementary Table 1).

Plants of ex situ collections were established from seeds provided by seed banks at the Botanic Garden Meise (Belgium), Freie Universität Berlin (Germany), Institut de Recherche pour la Conservation des Zones Humides Méditerranéennes (France), Millenium Seed Bank (UK), Museum National d’ Histoire Naturelle (France) Universidad Nacional del Sur (Argentina), and University of Sussex (UK). For establishing experimental collections, seeds were germinated under controlled conditions, in a growth chamber (Phytotron, Rumed) with a photoperiod of 18 h / 6 h of light and dark, respectively, and a temperature of 25 °C until germination [65]. Seedlings were transferred to jiffy pots and maintained in similar growth conditions for 2 months. After this period, they were transplanted into plastic pots with substrate and grown under greenhouse conditions for 1 year as described in [65].

### Library preparation and sequencing

Genomic DNA was extracted from liquid nitrogen grounded leaves of all samples collected for this work using the kit “innuPREP Plant DNA Kit” (Analytik Jena AG) with the SDS based Lysis Solution OPT protocol, which produced the best results for *Limonium* species. DNA was quantified in Qubit 2.0 (Invitrogen) using Qubit dsDNA HS Assay kit, quality checked using a Nanodrop 1000 (Thermo Fisher Scientific, Wilmington, DE, United States), and integrity verified using agarose gel electrophoresis (1%). Isolated DNA samples with a minimum concentration of 30 ng/μl were sent to Elshire GBS services (The Elshire Ltd., Manawatu, New Zealand) to perform library preparation and sequencing. Library preparation for deep sequencing was carried out using the TruSeq Nano DNA Library Preparation Kit (350 bp insert size). The genotyping-by-sequencing data was generated following the method described in [26] and included the following changes: 100 ng of genomic DNA were used, 3.6 ng of total adapters were used, genomic DNA was restricted with ApeKI enzyme and the library was amplified with 18 PCR cycles. Sequencing of *Limonium* cDNA libraries was carried out using the Illumina HiSeq Ten platform with 2 × 150 bp paired end reads.

### Raw data handling

Raw, paired end data was demultiplexed using axe-demux v0.3.3-2 [66]. The resulting demultiplexed “fastq” files were submitted to NCBI’s Sequence Read Archive (SRA) as “BioProject” PRJNA592300. These were then analysed using ipyrad v0.9.71 [67], using a “conda” [68] environment containing muscle v3.8.1551 [69] and vsearch v2.17.0 [70]. Read “assembly” was performed on two distinct sets of individuals. One comprised of all sampled sites and species (*L. brasiliense*, *L. californicum*, *L. carolinianum*, *L. humile*, *L. maritimum*, *L. narbonense* and *L. vulgare*) from here on designated as “species”, and a second one, comprising all *L. vulgare* and *L. maritimum* individuals, called “lvu_lma”. In both cases, the same assembly parameters were used, including GBS as *datatype*, a *clustering threshold* of 0.85, and a *mindepth* of 8. Other parameters were left at their defaults. In the “species” dataset, each species had to be represented by at least one individual for a SNP to be called. This restriction was not enforced on the “lvu_lma” dataset. Furthermore, in order to ensure only nuclear DNA was included in the analyses, a mitochondrial genome of *Beta vulgaris* (NCBI Accession BA000009.3) and a plastidial genome of *Limonium sinense* (NCBI Accession MN599096.1) were used to “bait” and discard mtDNA and cpDNA sequence reads. Full assemblies’ parameters can be found in a gitlab repository [71].

### Data analyses

Analyses downstream of the assembly were automated using “GNU Make” (https://www.gnu.org/software/make) to centralizing all details of each analysis step. To facilitate reproduction, a “ready to use” docker image [72] with all the software, configuration files, parameters, and the *Makefile*, is also provided. Furthermore, a gitlab repository [71] was created to host all files (and change history) necessary for analyses reproduction, such as the *Makefile*, static analysis parameter files, and a *Dockerfile*, (containing instructions on how to build the static analysis environment). Availability of the analyses’ environment (and creation process) should ensure that this work remains reproducible for the foreseeable future. This automation is an improvement upon a previous, similar scheme described in [73].

### Raw data filtering

Assembled data was filtered using VCFtools v0.1.14 [74]. Filtering parameters include, in this order: maximum percentage of missing data an individual can have in order not to be discarded (40 and 10% for the “species” “lvu_lma” datasets respectively); minimum number of individuals each SNP has to be represented in (80%); minimum allele frequency (MAF) of each SNP was set to 0.01 (any position where the rarer allele frequency is below 1% was discarded) – following the recommendations present in [46] to minimize population structure analyses bias.

In order to minimize the effects of linkage disequilibrium, the python script vcf_parser.py [75] as of commit “478a8e3” was used to keep only the SNP closest to each locus’s centre.

Phylip formatted files for phylogenetic analyses were filtered to include the same individuals that passed the above-mentioned filtering criteria.

PGDSpider v2.1.1.5 [76] was used to perform most file format conversions. Those not supported by PGDSpider were performed using scripts available in https://github.com/CoBiG2/RAD_Tools/ (technical details on these conversions can be found in the *Makefile*).

### Phylogenetic analyses

Phylogenetic analysis was conducted using RAxML v8.2.12 [77], *PTHREADS* version, using the model *GTRCAT*, and an automatic number of rapid bootstrap replicates. Seed value for both parsimony and rapid bootstrap inference was 112,358. It is important to note that there is no outgroup in this analysis, and as such, the tree rooting is artificial and arbitrary (chosen based on branch size, optimized for visualization).

### Ordination and clustering analyses

Two methods were used for grouping samples in order to understand the general pattern of individual or population structure, namely, Principal Components Analysis (PCA) and ALStructure (as of commit “e355411”) [78] – a likelihood-free, PCA based method to infer admixture plots. This method was chosen over the more popular likelihood based methods, such as STRUCTURE [79] due to the nature of polyploid data, which without phase information may bias likelihood model based methods.

The PCA was performed with the script snp_pca_static.R [75] as of commit “a69e9bd”.

To interpret admixture analyses results, an estimate of the optimal value of “K”, which represents how many demes the data can be clustered into is usually calculated. However, since ALStructure is PCA based, it can only produce such estimate in the absence of missing data. Since both used datasets have at least a fraction of missing data, an estimate of “K” was not possible to obtain. ALStructure was wrapped under Structure_threader v1.3.9 [80] and was run for values of “K” between 1 and 8.

### *L. vulgare* and *L. maritimum* differentiation

In order to further understand the genetic basis behind the *L. vulgare* and *L. maritimum* differentiation, SNPs fully segregated between these two species (using the “lvu_lma” dataset) were identified using the script segregating_loci_finder.py [75] as of commit “146b483”. Each segregated SNP’s flanking region was then obtained using the script loci_consensus.py [75] as of commit “d277ed3”. Since the obtained sequences are relatively small (between 41 and 261 bp), these sequences were queried against the *Limonium bicolor* transcriptome (GenBank: GBRK00000000.1) [81], using NCBI BLAST+ v2.9.0 [82] and for any relevant hits (*e-value* ≤ 1e^− 8^) the GBS locus was replaced with the transcriptome matched sequence. Resulting sequences where then queried against the UniParc database (release 2022_01) using DIAMOND v2.0.15 [83] and against NCBI’s “nt” database (as of 10-05-2022) using NCBI BLAST+. Only relevant hits (*e-value* ≤ 1e^− 8^) with plant sequences were considered valid matches.

## Supplementary Information

**Additional file 1: Supplementary Table 1.** List of specimens.

**Additional file 2: Supplementary Table 2.** Seed provenance.

**Additional file 3: Supplementary Table 3.** List of GBS loci hits to public databases.

## Data Availability

The datasets supporting the conclusions of this article are available in the NCBI SRA repository, under “BioProject” PRJNA592300. All analyses details and scripts are contained in a gitlab repository (dynamic) and in a zenodo repository(static, with DOI). A docker image containing the previous repository and all the required software, including usage instructions is available in docker hub. Filtered data can be found in this second zenodo repository.
