# Correction: A first look at sea-lavenders genomics – can genome wide SNP information tip the scales of controversy in the *Limonium vulgare* species complex?

**DOI:** 10.1186/s12870-023-04095-0

**Published:** 2023-02-04

**Authors:** Francisco Pina-Martins, Ana D. Caperta, Sofia I. R. Conceicao, Vera L. Nunes, Isabel Marques, Octavio S. Paulo

**Affiliations:** 1 cE3c - Centre for Ecology, Evolution and Environmental Changes & CHANGE -Global Change and Sustainability Institute, Departamento de Biologia Animal Faculdade de Ciencias, Universidade de Lisboa, Campo Grande, 1749-016 Lisbon, Portugal; 2 LEAF—Linking Landscape, Environment, Agriculture and Food, Associated Laboratory TERRA, Instituto Superior de Agronomia (ISA), Universidade de Lisboa, Tapada da Ajuda, 1349-017 Lisbon, Portugal; 3 LASIGE Computer Science and Engineering Research Centre, Faculdade de Ciencias, Universidade de Lisboa, 1749-016 Lisbon, Portugal; 4 Forest Research Centre (CEF) & Associated Laboratory TERRA, Instituto Superior de Agronomia (ISA), Universidade de Lisboa, 1349-017 Lisbon, Portugal

**Correction: BMC Plant Biol 23, 34 (2023)**

**https://doi.org/10.1186/s12870-022-03974-2**

Following the publication of the original article [1], the authors would like to update the **Funding** section.

The current Funding reads:

This work was supported by Portuguese national funds through Fundacao para a Ciencia e a Tecnologia (www. fct. pt/) projects PTDC/AGRPRO/4285/2014, LEAF UI/AGR/04129/2013 and UIDB/00329/2020 (study design, library preparation and sequencing), grants PTDC/AGRPRO/4285/BD/2014 (data analysis and manuscript writing), attributted to FPM, and PTDC/AGRPRO/4285/BM/2014 and PhD Scholarship ref. UI/BD/153730/2022 (wet-lab work) attributed to SIRC. The Linnean Society of London through the Percy Sladen Memorial Fund provided a grant to ADC for fieldwork in the project ‘Taxonomic reassessment of *Limonium* spp. (Plumbaginaceae) from Morocco Atlantic coast’. Computational resources were partially provided by INCD funded by FCT and FEDER under the project 01/SAICT/2016 n° 022153.

The correct Funding should read:

This work was supported by Portuguese national funds through Fundacao para a Ciencia e a Tecnologia (www. fct. pt/) projects PTDC/AGRPRO/4285/2014, LEAF UI/AGR/04129/2013 and UIDB/00329/2020 (study design, library preparation and sequencing), grants PTDC/AGRPRO/4285/BD/2014 (data analysis and manuscript writing), attributted to FPM, and PTDC/AGRPRO/4285/BM/2014 and PhD Scholarship ref. UI/BD/153730/2022 (wet-lab work) attributed to SIRC **UIDB/00239/2020 (CEF), under the Scientific Employment Stimulus—Individual Call (CEEC Individual)—2021.01107.CEECIND/CP1689/CT0001 (IM).** The Linnean Society of London through the Percy Sladen Memorial Fund provided a grant to ADC for fieldwork in the project ‘Taxonomic reassessment of *Limonium* spp. (Plumbaginaceae) from Morocco Atlantic coast’. Computational resources were partially provided by INCD funded by FCT and FEDER under the project 01/SAICT/2016 n° 022153.

The correction does not have any effect on the results or conclusions of the paper. The original article [1] has been corrected.
