# Supplementary material for: A first look at sea-lavenders genomics – can genome wide SNP information tip the scales of controversy in the Limonium vulgare species complex?
Source: BMC Plant Biol. 2023 Jan 16;23:34. doi: 10.1186/s12870-022-03974-2 (PMC9841708; doi:10.1186/s12870-022-03974-2)
Supplement: Supplementary file 1 — Additional file 1: Supplementary Table 1. List of specimens. [file 12870_2022_3974_MOESM1_ESM.docx]

**Supplementary Table 1.** List of specimens used in genomic studies sampled in France, Morocco, Portugal, Spain and Sweden. Individuals from populations marked with an asterisk were previously analysed for ITS sequence variation in A. S. Róis et al. (2018). Representative specimens of the populations are deposited in the Herbarium João de Carvalho e Vasconcellos (LISI) at Instituto Superior de Agronomia, Lisbon, Portugal. Location names in **boldface** font represent individuals that were sampled from germinated seeds. Samples without voucher number represent field plants and no herbarium specimens exist from such plants.

| **Species** | **Population origin** | **Geographical coordinates** | | **Voucher number** | **Number of specimens analysed** |
| --- | --- | --- | --- | --- | --- |
| ***L. humile*** | Sweden: Styrsö, Amnevik | 57° 36′ 42.8″N | 11° 46′ 14.6″E | — | 3 |
| ***L. maritimum*** | Portugal: Viana do Castelo, Areosa* | 41°43′14.6″N | 8° 52′ 1.9″W | LISI — 134/2012 | 7 |
|  |  |  |  |  |  |
| ***L. narbonense*** | Spain: Alicante, Denia, Dehesas* | 38° 52′ 48.9″N | 0° 2′ 8.1″W | LISI — 318/2016 | 3 |
|  | SP: Castellon, Torre de la Sal, Prat de Cabanes* | 40°8′16.1″N | 0° 9′ 56.9″E | LISI — 321/2016 | 3 |
|  |  |  |  |  |  |
| ***L. vulgare*** | Morocco: Larache, Moulay Bousselham* | 34° 52′ 25.4″N | 6° 17′ 12.9″W | LISI — 723/2015 | 6 |
|  | **Portugal: Aveiro, Boco*** | 40° 58′ 94.8″’N | 8° 68′ 79.7″’W | LISI — 121/2012 | 1 |
|  | **Portugal: Aveiro, Ribeira da Aldeia*** | 40° 47′ 43.8″N | 8° 37′ 47.5″W | LISI — 161/2012 | 4 |
|  | **Portugal: Ílhavo, Gafanha do Carmo** | 40° 36′ 25.6″N | 8° 44′ 9.7″W | LISI — 120/2012 | 2 |
|  | **Portugal: Odemira, São Luís, Casa Branca*** | 37° 66′ 63.4″N | 8° 72′ 00.9″W | LISI — 113/2012 | 4 |
|  | **Portugal: Setúbal, Amora** | 38° 62′ 67.0″N | 9° 1′ 60.2″W | — | 2 |
|  | **Portugal: Setúbal, Barreiro** | 38° 61′ 67.6″N | 9° 04′ 75.7″W | — | 1 |
|  | **Portugal: Setúbal, Mouriscas*** | 38° 52′ 84.4″N | 8° 80′ 43.5″W | LISI — 139/2012 | 2 |
|  | **Portugal: Setúbal, Alcochete, Sítio das Hortas*** | 38° 76′ 04.4″N | 8° 93′ 74.1″W | LISI — 127/2012 | 3 |
|  | **Portugal: Tavira, sapal do Barril*** | 37° 05′ 26.2″N | 7° 40′ 21.8″W | — | 6 |
|  | Spain: A Coruña, Ortigueira, Morouzos Playa Caballar | 43° 42′ 17.6″N | 7° 51′ 26.1″W | LISI — 857/2017 | 2 |
|  | Spain: Huelva, Ayamonte | 37° 12′ 41.20″N | 7° 23′ 25.84″W | — | 7 |
|  | Sweden: Styrsö, Brännholmsviken | 57° 36′ 07.5″N | 11° 46′ 34.2″E | — | 2 |
