# Supplementary material for: A first look at sea-lavenders genomics – can genome wide SNP information tip the scales of controversy in the Limonium vulgare species complex?
Source: BMC Plant Biol. 2023 Jan 16;23:34. doi: 10.1186/s12870-022-03974-2 (PMC9841708; doi:10.1186/s12870-022-03974-2)
Supplement: Supplementary file 2 — Additional file 2: Supplementary Table 2. Seed provenance. [file 12870_2022_3974_MOESM2_ESM.docx]

**Supplementary Table 2.** **Seed provenance of plants used in the genomic studies.** Plants were originated from seeds obtained from seed banks and living plants were maintained in the greenhouses at Instituto Superior de Agronomia (ISA), Lisbon, Portugal. All accessions are identified with the unique identification number of the accession as used by the respective Seed Bank. Information on species is given in alphabetical order. Abbreviations: AR – Argentina; BE – Belgium; FR – France; GER – Germany, UK – United Kingdom; and USA – United States of America.

| **Species** | **Seed accession number** | **Seed provider** | **Origin** | **Number of individuals analysed** |
| --- | --- | --- | --- | --- |
| *L. brasiliense* | UNS1-2017 | Universidad Nacional del Sur, Bahía Blanca | AR: Buenos Aires, Bahía Blanca | 5 |
| *L. carolinianum* | 569691 | Millenium Seed Bank, Kew | USA: Texas, Brazoria County | 4 |
| *L. carolinianum* | 573276 | Millenium Seed Bank, Kew | USA: Massachusetts | 1 |
| *L. californicum* | 440844 | Millenium Seed Bank, Kew | USA: California | 3 |
| *L. narbonense* | JB 71078 | Seed Bank, Museum National d'Histoire Naturelle, Paris | FR: Languedoc Roussillon, Pyrénées Orientales, Toreilles | 4 |
| *L. narbonense* | IRCZH1-2017 | Institut de Recherche pour la Conservation des Zones Humides Méditerranéennes | FR: Bouches-du-Rhône, Camargue, Tour du Valat | 3 |
| *L. vulgare* | 78162 | Millenium Seed Bank, Kew | UK: South East, Isle of Wight | 4 |
| *L. vulgare* | 123811 | Millenium Seed Bank, Kew | UK: West Sussex | 3 |
| *L. vulgare* | 19892063 | Seed Bank, Botanic Garden Meise, Brussels | BE: West-Vlaanderen, Nieuwpoort, Nature reserve "De Ijzemonding" | 4 |
| *L. vulgare* | 195.01.12.14 | Dahlem Seed Bank, Freie Universität Berlin | GER: Schleswig - Holstein, Salzwiese | 1 |
| *L. vulgare* | JB 74321 | Seed Bank, Museum National d'Histoire Naturelle, Paris | FR: Brittany, Morbihan, Le Hézo | 3 |
| *L. vulgare* | JB 67362 | Seed Bank, Museum National d'Histoire Naturelle, Paris | FR: Pays de la Loire, Vendée, Brem-sur-Mer | 2 |
