# Supplementary material for: A first look at sea-lavenders genomics – can genome wide SNP information tip the scales of controversy in the Limonium vulgare species complex?
Source: BMC Plant Biol. 2023 Jan 16;23:34. doi: 10.1186/s12870-022-03974-2 (PMC9841708; doi:10.1186/s12870-022-03974-2)
Supplement: Supplementary file 3 — Additional file 3: Supplementary Table 3. List of GBS loci hits to public databases. [file 12870_2022_3974_MOESM3_ESM.docx]

**Supplementary Table 3.** List of GBS loci matched to *L. bicolor*’s transcriptome, Uniparc and nt. Relvant plant hits are described in the “Function” column (or just marked as “Not a plant hit”, if the sequence was matched to a non-plant organism).

| **Locus** | **Transcriptome match** | **Uniparc Match** | **nt Match** | **Function** | **E-value** |
| --- | --- | --- | --- | --- | --- |
| vcf_locus984 | NA | UPI00053F3CAE |  | [Reverse transcriptase domain](https://www.ebi.ac.uk/interpro/entry/InterPro/IPR000477/) | 8.66e-08 |
| vcf_locus3701 | NA | NA |  | NA | NA |
| vcf_locus3813 | NA | UPI00053F376D |  | [Pentatricopeptide repeat](https://www.ebi.ac.uk/interpro/entry/InterPro/IPR002885/) | 9.01e-06 |
| vcf_locus4045 | NA | NA |  | NA | NA |
| vcf_locus4214 | GBRK01021417.1 | UPI000B76F5A5 |  | [Transcription factor, TCP](https://www.ebi.ac.uk/interpro/entry/InterPro/IPR005333/) | 1.42e-19 |
| vcf_locus12565 | NA | UPI00053FE0B0 |  | [Phospholipase C, phosphatidylinositol-specific, Y domain](https://www.ebi.ac.uk/interpro/entry/InterPro/IPR001711/) | 2.01e-08 |
| vcf_locus16230 | NA | NA |  | NA | NA |
| vcf_locus16406 | NA | NA |  | NA | NA |
| vcf_locus31470 | NA | UPI000A379FE2 |  | Not a plant hit | 2.34e-09 |
| vcf_locus35500 | NA | NA |  | NA | NA |
| vcf_locus46709 | GBRK01023424.1 | UPI00085CEB18 |  | [Proton-dependent oligopeptide transporter family](https://www.ebi.ac.uk/interpro/entry/InterPro/IPR000109/) | 6.23e-121 |
| vcf_locus46859 | NA | NA |  | NA | NA |
| vcf_locus47182 | NA | NA |  | NA | NA |
| vcf_locus47254 | NA | NA |  | NA | NA |
| vcf_locus52961 | NA | NA |  | NA | NA |
| vcf_locus56129 | NA | NA |  | NA | NA |
| vcf_locus58499 | NA | NA |  | NA | NA |
| vcf_locus59370 | NA | NA |  | NA | NA |
| vcf_locus80371 | NA | NA |  | NA | NA |
| vcf_locus83110 | NA | UPI0019551739 |  | Not a plant hit | 8.35e-15 |
| vcf_locus83229 | NA | NA |  | NA | NA |
| vcf_locus83781 | NA | NA |  | NA | NA |
| vcf_locus87444 | NA | NA |  | NA | NA |
| vcf_locus95053 | GBRK01036747.1 | UPI001C2AC88F |  | [Zinc finger, PHD-type](https://www.ebi.ac.uk/interpro/entry/InterPro/IPR001965/) | 1.36e-122 |
| vcf_locus97528 | NA | NA |  | NA | NA |
| vcf_locus98466 | NA | NA |  | NA | NA |
| vcf_locus101857 | NA | NA |  | NA | NA |
| vcf_locus106586 | NA | NA |  | NA | NA |
| vcf_locus112571 | NA | NA |  | NA | NA |
| vcf_locus116158 | NA | NA | CP020100.1 | Not a plant hit | 3.00e-04 |
| vcf_locus128709 | NA | NA |  | NA | NA |
| vcf_locus133950 | NA | NA |  | NA | NA |
| vcf_locus138379 | NA | NA |  | NA | NA |
| vcf_locus140215 | NA | NA |  | NA | NA |
